# Diet and nutritional status among hospitalised children in Hawassa, Southern Ethiopia

**DOI:** 10.1186/s12887-022-03107-6

**Published:** 2022-01-21

**Authors:** Vilde K. Hjellbakk, Hailu Hailemariam, Fikadu Reta, Ingunn M. S. Engebretsen

**Affiliations:** 1 Centre for International Health (CIH), Department of Global Public Health and Primary Care (IGS), Faculty of Medicine and Dentistry, University of Bergen, Bergen, Norway; 2 School of Nutrition, Food Science and Technology, Hawassa University, Hawassa, Ethiopia

**Keywords:** Nutrition, Severe acute malnutrition, Stunting, Wasting, Dietary patterns, Ethiopia, Children under 5 years of age

## Abstract

**Background:**

Undernutrition constitutes a major problem among children in Hawassa, Ethiopia, and the literature on nutritional status in hospitalised children is scarce. The aim of this study was to investigate dietary diversity, nutritional practices, and the frequencies of undernutrition and the factors associated with severe acute malnutrition (SAM) in a hospitalised paediatric population in Hawassa, Southern Ethiopia.

**Methods:**

A hospital-based cross-sectional study was carried out among hospitalised children in Hawassa, Southern Ethiopia. Children aged 6 to 59 months and their caregivers admitted for >24 hours from two public hospitals in Hawassa between November 2019 and January 2020 were included. Dietary diversity was assessed using World Health Organization (WHO) guidelines. Weight and height/length of the children were measured, and z-scores were calculated using the WHO growth standards. The definition of SAM was a weight-for-height z-score (WHZ) less than –3 or a clinically confirmed SAM diagnosis with higher WHZ.

**Results:**

A total of 188 caregiver-child pairs were assessed in the two public hospitals. The majority of the patients were admitted with SAM (*N* = 70/188, 37%) or respiratory tract infections (N = 44/188, 23%). There was a similar number of boys and girls with SAM. Of all the children, 59% reported to have consumed foods from fewer than four food groups, while 40% reported eating foods from four or more food groups. The rate of malnutrition was high, and 35.8% of the children were classified as wasted (WHZ < –2) and 41% were stunted (height-for-age z-score < –2). Nearly 30% of the SAM patients were also stunted.

**Conclusion:**

This study revealed that hospitalised children in this setting had poor dietary diversity and nutritional status, a high degree of morbidity, and extreme poverty. There is thus a need to focus on nutrition patterns in clinical settings.

**Supplementary Information:**

The online version contains supplementary material available at 10.1186/s12887-022-03107-6.

## Background

According to the Ethiopian health survey, only 14% of Ethiopian children meet the recommended level for diet diversity, defined as eating foods from four out of seven specified groups in the last 24 hours before the survey [1]. On a national level, 38% of children under the age of 5 years are stunted (height-for-age z-scores (HAZ) < –2) and 18% are severely stunted (HAZ < –3). The Southern Nations, Nationalities, and People’s Region (SNNPR) is close to the national average on undernutrition parameters [1]. Ten percent of Ethiopian children are wasted (weight-for-height (WHZ) < –2), and 3 % are severely wasted (WHZ < –3). Wasting and stunting are often considered as separate outcomes of undernutrition and are managed differently for both treatment and prevention [1], although newer research emphasizes their interconnectedness [2]. Wasting, also known as ‘acute malnutrition’, is associated with increased mortality and morbidity and long-term mental and somatic impairment and requires urgent attention [3].

In 2019, the Federal Ministry of Health Ethiopia developed national guidelines for management of acute malnutrition based on the revised World Health Organization (WHO) guidelines as implemented in the SNNPR [4]. Major efforts have been established to identify and treat children in the communities and primary health care facilities. Ethiopia has adapted the Community Management of Acute Malnutrition (CMAM) approach to screen children under the age of 5 years for severe acute malnutrition (SAM) and to treat moderate acute malnutrition using ready-to-use supplementary foods or a fortified blended food [4]. Children with SAM defined by mid-upper-arm circumference (MUAC) less than 115 mm or any clinical symptoms such as oedema are referred to a health facility for further assessment and treatment [5].

In the health care facilities, MUAC, WHZ, or weight-for-length (WLZ) status is checked. Children aged 6–59 months who have bilateral oedema, a MUAC below 115 mm, or a WHZ/WLZ below –3 SD (standard deviation) according to the WHO growth standards should be treated in a SAM program [5]. If the children have SAM, they are classified as complicated or uncomplicated cases [6]. Children who pass the appetite test and are clinically well (uncomplicated cases) are usually treated at the outpatient clinic with weekly visits to a health post or facility. They will receive ready-to-use supplementary foods so that the family or caregivers can manage them at home [6]. If the child fails the appetite test or has severe oedema, other medical complications, or one or more of the Integrated Management of Childhood Illness warning signs, they should be admitted to a clinic or hospital [5, 6]. The children can later be transferred to outpatient treatment when the medical complications are resolved and their appetite is back [5, 6]. To our knowledge, the implementation of the updated guideline has not been fully evaluated yet.

Although the CMAM programs involve hospitals for referrals, the literature on nutritional status in hospitalised children is scarce. A cross-sectional study from Gondar, Northwest Ethiopia, reported that among hospitalised children aged 6 months to 15 years, 21.6% were classified as malnourished, and nearly half of these were severely malnourished [7].

In addition, nutritional practices before and during admission are not known. This study assessed dietary diversity, nutritional practices, the frequencies of undernutrition, and the socio-demographic factors associated with SAM in a hospitalised paediatric population in two hospitals in Hawassa in the Sidama region of Ethiopia.

## Method

### Study Area - Hawassa, Sidama Region, Ethiopia

The study was conducted in the city of Hawassa, which before November 2019 served as an administrative city for the SNNPR. In mid-November 2019, the Sidama referendum was held to vote for a new Sidama region, separating from the SNNPR. The city is approximately 1700 to 1900 m above sea level, with an average annual rainfall of 1090 mm. The wet season is from March to October with a dryer shorter season from November to February. Hawassa has an estimated 350,000 inhabitants. The city has one public specialized teaching hospital (Referral University Hospital), one public general hospital (Adare Hospital), and four public health centres. The national guideline for the management of acute malnutrition had been implemented at both Adare Hospital and Referral University Hospital.

### Study participants, design, and period

The study was conducted as a hospital-based cross-sectional study and lasted from November 2019 to January 2020, corresponding to the dryer season. The target group for this study was children aged 6.0–59.9 months admitted to either the Adare Hospital or Hawassa University Hospital for more than 24 hours. Both hospitals are public, government hospitals in Hawassa city, and children come from the surrounding districts and mainly from the Sidama and Oromia regions. The recruitment of participants and data collection were performed concurrently over the study period. Child-caregiver pairs were not eligible for participation if the child had a terminal illness, if the child already had participated in any intervention trials at an earlier age, or if the child and/or guardian were not able to collaborate (i.e., any somatic or mental disability making collaborations more difficult than expected for their age range),

### Sample size

The sample size was calculated using Open Source Epidemiologic Statistics for Public Health [8], and the sample size for proportion was used assuming 95% confidence levels and 10% confidence limits. The Ethiopian Demographic and Health Survey (EDHS) from 2016 reported the prevalence of stunting in the SNNPR to be 39% for children under the age of 5 years, and the anticipated frequency was therefore set to 39%. Stunting was chosen as a factor because it represents children with impaired growth and development from poor nutrition, inadequate psychosocial stimulus, and recurrence of infections [9]. The sample size was calculated to be 92 participants from both hospitals. Considering a 10% non-response rate, the sample size was adjusted to 100 children per hospital for 200 child-carer pairs in all.

### Ethical considerations

Ethical clearance was obtained from the Institutional Review Board at the College of Health Science, Hawassa University (Ref. No IRB/262/12, date: 28.10.2019), and the Regional Committee for Medical and Health Research Ethics of Western Norway (REK Vest, no. 32315, date: 07.10.2019). Caregiver-child pairs providing informed consent were eligible for participation.

A letter with information about the study in Amharic was provided for participants and was explained to eligible participants by trained supervisors. Participants/caregivers could at any time withdraw from the study without negative consequences. The participants did not get any incentives for participation but were informed about how their participation and findings might inform researchers and benefit the improvement of children’s health in the future.

### Data collection

A structured pretested questionnaire was used to collect data through a face-to-face interview. Data collectors were staff and postgraduate students from the Nutrition Department at Hawassa University. There were six data collectors, three in each hospital. The gender balance was two male and one female data collector in the Referral University Hospital and two female and one male data collector in the Adare Hospital. In addition, one paediatric nurse who was working in the hospital served as a supervisor in each hospital.

Both the data collectors and supervisors recruited participants, conducted the interviews, and performed the anthropometric measurements. The supervisors also oversaw raw data management and storage and answered medical questions from the data collectors. They had the responsibility for ensuring the correct clinically given medical diagnosis in the questionnaire. All data collectors and supervisors spoke Amharic in addition to the most common languages in this area, namely Wolaytta, Afan, Oromo, and Sidamu Afo. Nurses, doctors, and other staff served as translators if needed.

### Instruments and translations of tools

Caregivers of the included children were interviewed using a pretested, structured, standardized questionnaire in the local languages, and the caregivers were asked to respond to the best of their knowledge. The questionnaire was developed in English and further translated into Amharic. Back-translation and content validation were performed to ensure the best translation possible.

### Anthropometry

The anthropometric measurements included height/length, weight, and MUAC. Two readings were made for each measurement and an average was calculated. This was used to assess the child’s nutritional status. MUAC was measured with a standard MUAC tape. Weight was measured with a baby-weight scale for children under 2 years of age. Children above 2 years were measured on a Seca weighing scale. If the child above 2 years was unable to stand, tared weight was measured. Recumbent length was measured for children under 24 months with a length-board with a sliding headpiece, and height was measured for children aged 24–59 months with a stadiometer according to WHO standards [10]. The data collector had training in anthropometric measurements, and additional training was given to ensure that standard procedures were followed. Length/height was given to the nearest 0.1 cm, and weight was given to the nearest 0.1 kg.

### Data entry, cleaning, and analysis

EpiData Manager and EpiData Entry Client version 4.6 were used for data entry. Double entry was done for only 16% of the forms. There were mistakes found in 10 records and 15 fields (variables), equivalent to a field error rate of 0.05%. The data were exported to STATA version 14 (StataCorp, Texas, USA) for data management and subsequent analysis.

Ethiopia has its own calendar, and multiple studies have shown that the responding caretakers do not usually remember the birth date of their baby and often report estimated ages. Because of this, the questionnaire gathered information on both ‘date of birth’ and ‘age in months’ in an attempt to quality check the age. The exact age of the participants was calculated when the birth date was available and compared to the given ‘age in months’. In cases where birth month and year were given but not the date, the date of birth was set to the median of the month. The discrepancy between the ‘age in months’ and the estimated age based on the date of the interview and the date of birth was calculated. If the discrepancies were more than +/–2 months, the age was set to missing. The reported ‘age in months’ and the calculated age in months based on the date of the interview and ‘date of birth’ were in agreement in 85% of the children. Z-scores based on age were therefore calculated for this group. Participants who did not report date of birth, only age in months, were not excluded from HAZ and weight-for-age z-score (WAZ) analysis.

Calculation of z-scores was done in STATA by using the WHO Child Growth Standards (see Supplementary file for details). Anthropometric data were first checked for outliers.

### Clinical diagnoses

The medical diagnoses were recorded as text answer options, and the supervisors (or other medical staff) entered the primary diagnoses of the patients. In a few cases, when the supervisors and hospital staff were not available, the medical diagnosis was given by the caregiver according to what they had been told. All diagnoses were double-checked with admission papers and hospital records. After data entry, the diagnoses were aggregated into larger groups that included SAM, acute respiratory infections, abdominal infections, fevers/other infections, and other ailments. The fever/other infection group and the other group were later clustered together because they were small groups.

### Principal component analysis (PCA) index

A PCA-based index was used as a proxy measure of household socioeconomic status. To construct the PCA-based socioeconomic index (SE index), a subset of indicators including assets and wealth indicators was used. The final asset variables for analysis were having a radio, mobile telephone, television, computer, refrigerator, mitad (electric oven), and motorbike and ownership of domestic animals such as cattle, sheep/goats, donkeys, and chickens. In addition, health and hygiene indicators such as water source and treatment and toilet facilities were analysed. First, descriptive statistics were assessed for all the variables. The PCA was performed in STATA with the pca-command and the minimum eigenvalue set to 0.5. The first principal component scores were used, and the first component explained 26% of the variance. The resulting asset index was divided into three groups for further analysis. In order to assess the content validation, the distribution of assets was checked against the three groups, as suggested by the MRC South Africa [11].

### Dietary diversity score (DDS)

Food consumed by the children 7 days before admission to the hospital was classified into the following seven food groups based on recommendations from the WHO: 1) cereals, roots, and tubers, 2) legumes and nuts, 3) dairy products, 4) flesh foods such as meat, fish, and poultry, 5) eggs, 6) vitamin A-rich fruits and vegetables, and 7) other fruits and vegetables [12].

A DDS was defined as the number of food groups consumed and was used to measure the dietary diversity of children before admission. The information was based on the dietary data collected from primary caregivers, and the DDS was calculated in STATA by summarizing the food groups. Every food consumed from different groups was marked as 1 point, and eating several foods within one group would still only count for 1 point. The maximum number of points was seven, one for each group. Minimum dietary diversity is when four food groups are consumed, and having a score less than four indicates low dietary diversity. The method was originally developed for children aged 6–23 months of age and a 24-hour recall. Because many of the children already had been admitted to the hospital for some days, we chose to recall the diet the 7 days before admission. Because the sample size was small and we did not know how many children would be in the two different age groups (6–23 months old and 24–59 months old), an extrapolation was done and a DDS was calculated for the whole sample size.

### Household Food Insecurity Access Scale (HFIAS)

Household food insecurity access was measured, classified, and calculated according to the Food and Nutrition Technical Assistance Guideline [13]. The household food insecurity access score and prevalence was calculated from two types of related questions: occurrence and frequency. First, a question was asked about a specific condition associated with experiencing food insecurity over the last 4 weeks. If the participant’s answer was yes, a frequency question followed with response option of rarely, sometimes, or often. These options each had values, respectively, of 1, 2, and 3, which were used to calculate the household food insecurity access score. The lowest possible score was 0 and the highest was 27. A higher score represents more experience of food insecurity [13]. The household food insecurity access score and prevalence indicator was used to separate households into the following four categories of food insecurity (access): food secure, mildly food insecure, moderately food insecure, and severely food insecure [13].

### Statistical analyses

Descriptive statistics are presented as frequencies and percentages for categorical variables and as means and 95% confidence intervals (CIs) for continuous variables. This included socio-demographic characteristics as well as dietary information, household food insecurity, and growth indicators. Descriptive data for dietary diversity and the number of food groups consumed were illustrated graphically with the distribution of all scores from 0 to 7 in Fig. 2. Binary logistic regression was performed to identify associations between the dependent and independent variables. The dependent variable was a SAM diagnosis, where SAM = 1 and not SAM = 0. In addition to the children admitted with a SAM diagnosis, all children calculated to have a WHZ < –3 were included in the SAM variable. The independent variables were socio-demographic characteristics, DDS, household food insecurity, stunting, hospitalisation prior to admission, and symptoms of diarrhoea, pneumonia, or fever prior to admission. Collin-command was used to check for collinearity. All variables that were associated with the dependent variable in the binary logistic regression analysis with a p-value < 0.05 were included in the adjusted analysis model. The logistic regression analyses were done in STATA with the logistic-command, and cluster adjustment was performed using hospital as a unit. The level of statistical significance was set to a p-value < 0.05.

## Results

### Socio-demographic characteristics

A total of 188 caregiver-child pairs were interviewed in the two public hospitals Adare Hospital and Referral University Hospital in Hawassa between November 2019 and January 2020. The mean age for caregivers was 27 years, and the mean age for the children was 18 months (Table 1). The majority of the children (63%) were in the age group 6–23 months of age. The majority of the caregivers were mothers who were married (Table 1), and the most common religious practice was Islam. The majority of the mothers (85%) reported their education to be primary school or less, and 74% reported not having any paid work. The majority of fathers had primary and/or secondary schooling, 55% and 21%, respectively.

**Table 1:** Sociodemographic characteristics of patients, categorical and continuous data

Variable	Category	SAM *n* = 70 n (%)	Not SAM *n* = 118 n (%)	Total *n* = 188 n (%)
Gender	Female	33 (48.5)	83 (72.8)	116 (63.7)
	Male	35 (51.5)	31(27.2)	66 (36.3)
Hospital	Adare	7 (10.0)	66 (55.9)	73 (38.8)
	Referral	63 (90.0)	52 (44.1)	115 (61.2)
Relationship to child	Mother	61 (87.1)	110 (93.2)	171 (91.0)
	Other	9 (12.9)	8 (6.8)	17 (9.0)
Language (primary)	Amharic	5 (7.1)	22 (18.6)	27 (14.4)
	Oromic	35 (50.0)	20 (17.0)	55 (29.3)
	Sidamic	10 (14.3)	34 (28.8)	44 (23.4)
	Other	6 (8.6)	19 (16.1)	25 (13.3)
Region	SNNPR/Sidama	22 (31.4)	66 (55.9)	88 (46.8)
	Oromia	32 (45.7)	27 (22.8)	59 (31.4)
	Other	1 (1.4)	1 (0.9)	2 (1.1)
Religion	Orthodox	6 (8.6)	24 (30.3)	30 (16.0)
	Protestant	22 (31.4)	64 (54.2)	86 (45.7)
	Muslim	37 (52.9)	26 (22.0)	63 (33.5)
	Other	5 (7.1)	4 (3.4)	9 (4.8)
Marital status	Single	4 (5.9)	28 (23.9)	32 (17.3)
	Married	57 (83.8)	88 (75.2)	145 (78.4)
	Other	7 (10.3)	1 (0.8)	8 (4.3)
Mothers education	No school	29 (42.6)	37 (31.6)	66 (35.7)
	Primary	29 (42.6)	44 (37.6)	73 (39.5)
	Secondary	10 (14.7)	36 (30.8)	46 (24.9)
Mothers occupation	Unemployed	52 (74.4)	87 (73.7)	139 (74.3)
	Employed	17 (24.6)	31 (26.3)	48 (25.7)
Fathers education	No school	14 (24.1)	28 (24.4)	42 (24.3)
	Primary	32 (55.2)	29 (25.2)	61 (35.3)
	Secondary	12 (20.7)	58 (50.4)	70 (40.5)
Fathers occupation	Unemployed	42 (68.9)	56 (48.7)	98 (55.7)
	Employed	19 (31.1)	59 (51.3)	78 (44.3)
SE-index	“Lower index”	35 (50.0)	27 (23.7)	62 (33.7)
	“Middle index”	25 (35.7)	36 (31.6)	61 (33.2)
	“Higher index”	10 (14.3)	51 (44.7)	61 (33.2)
**Continuous data**		**n (mean)** **95% CI (SD)**		
Age of child in months		63 (18.2) 14.9 – 21.5 (12.9)	108 (17.8) 15.5 – 20-.2 (12.3)	171 (18.0) 16.1 – 19.8 (12.5)
Age of caregiver in years		69 (27.4) 25.5 – 29.3 (8.0)	113 (27.6) 26.5 – 28.7 (5.9)	182 (27,5) 26.6 – 28.5 (6.7)

*Abbreviations*: *SAM* Severe acute malnutrition, *CI* confidence interval, *SD* standard deviation

### Clinical presentation

**Fig. 1:**
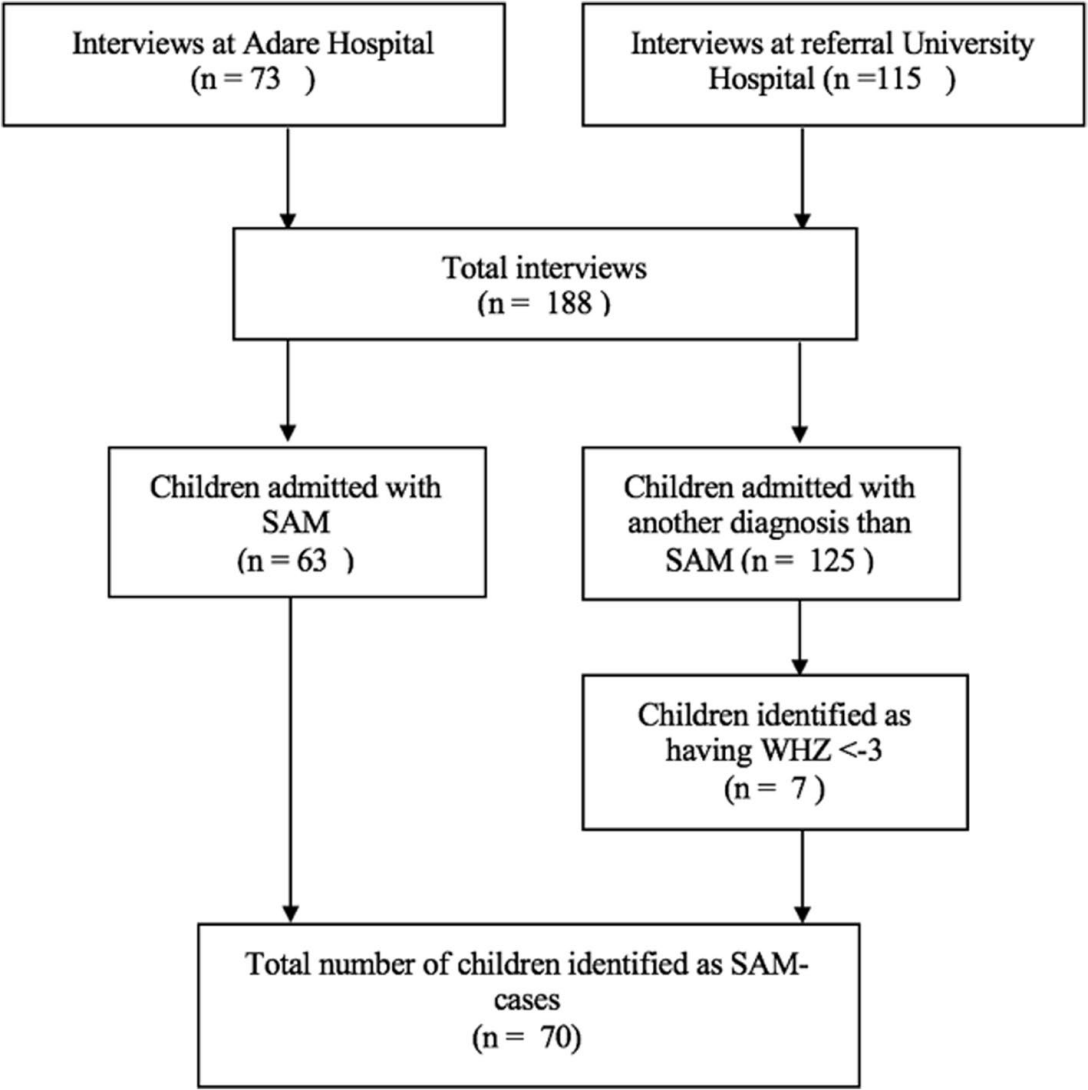
Identification of children with SAM from two hospitals in Hawassa and the clinical definitions

A total of 63 children were admitted with a SAM diagnosis, and an additional 7 children were found to have a WHZ < –3. Respiratory tract infections were the second-most common condition among the hospitalised children (Table 1 Supplementary file). In total 70 children were classified as having SAM (Fig. 1).

Of the children admitted with SAM, 63 children (90%) were admitted to Referral University Hospital and 7 (10%) children were admitted to Adare Hospital. The gender distribution for SAM was fairly equal with 33 (48.5%) children being female and 35 (51.5%) being male. The results of the SE-index indicated that the majority of the children admitted with SAM were in the lower socio-economic index category, meaning they experienced a greater degree of poverty than the other group.

### Dietary diversity

**Fig. 2:**
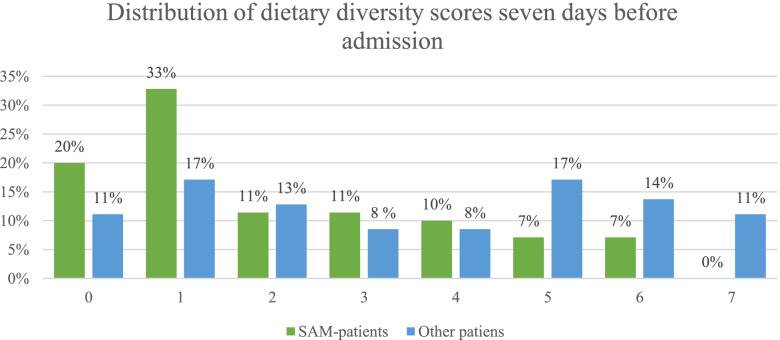
Distribution of dietary diversity scores seven days before admission

Figure 2 shows the distribution of the population's consumption of food groups, and frequencies and percentages are given indicating increasing numbers of food groups in the last 7 days prior to admission. A total of 59% reported having consumed foods from fewer than four food groups, while 40% reported eating foods from four or more food groups (Fig. 2).

Children with SAM had less dietary diversity than children without SAM in the last 7 days prior to admission, and only 25% of the children with SAM consumed foods from four groups or more groups compared to 50.4% among those not having SAM. The mean DDS was 2.1 (95%CI 1.6–2.5) for the children admitted with SAM compared to 3.5 (95%CI 3.1–3.9) among children without SAM. There was a difference in score between the two age groups of 6–23 months and 24–59 months. For the children aged 6–23 months admitted with SAM, the mean number of food group consumed was 1.9 (95%CI 1.3–2.5), and for children aged 24–59 months the mean score was 2.9 (95%CI 2.2–3.7). The corresponding numbers for children without SAM were 3.2 (95%CI 2.7–3.7) and 4.1 (95%CI 3.2–5.0), respectively.

Generally, consumption of other food groups was low. A clear difference was seen in consumption between the two patient groups, where SAM patients had lower consumption in all categories. Of the total sample size, 40% reported the child to have consumed eggs seven days prior to admission (Fig. 1 supplementary file). The category with lowest consumption in both patient groups was “flesh food”, and the category with greatest variation between the two patient groups was “other fruits and vegetables”.

### Household food insecurity access scale

The mean HFIAS score was calculated to be 4.7 (95%CI 3.8–5.5). The prevalence of different levels of household food insecurity (access) was calculated from 171 participants. The results showed that 50.9% were food secure, 2.3% were mildly food insecure, 15.8 % were moderately food insecure, and 31% were severely food insecure.

### Anthropometry

A total of 118 out of the 188 participants had information about both the age given by the caregiver and the date of birth. For 12 out of the 118 participants, birth month and year was given but not the exact day. Out of the 118, 16 participants had a discrepancy of more than +/–2 months between the reported age and the calculated age, and therefore the dates and ages were set to missing. Three participants had ages that were calculated to be younger than 6 months, but because they were within the +/–2 discrepancy criterion they were not excluded. Of all participants, 69 did not report date of birth, only the age in months. Based on the WHO flagging z-score criteria, the number (%) of participants excluded was 5 (3%) for WAZ, 17 (10.4%) for HAZ, 15 (9.2%) for WLZ, and 8 (4.8%) for MUAC z-score (MUACZ).

Table 2 shows the distributions of z-scores within each category of HAZ, WAZ, WHZ, and MUACZ. According to the results, 40.1% of the children were classified as stunted, of which half of them were severely stunted, 35.8% of the children were classified as wasted, of which 60% were severely wasted, and more than 60% of the children were underweight. According to the MUACZ, 19% of the children were moderately malnourished and 29% were severely malnourished.

**Table 2:** Average Z-score values and distribution of z-scores in different categories

	HAZ ***n*** = 146	WAZ ***n*** = 160	WHZ/WLZ ***n*** = 148	MUACZ ***n*** = 157
**Mean** **(95% CI)**	-1.4 (-1.8; -1.0)	-1.6 (-2.0; -1.3)	-0.9 (-1.3; -0.5)	-2.1 (-2.3; -1.9)
**Undernutrition**	**Stunting** **n (%)** 60 (40,1)	**Underweight** **n (%)** 99 (61,9)	**Wasting** **n (%)** 53 (35,8)	**Malnutrition** **n (%)** 76 (48,4)
**z-score groups:**	**n (%)**	**n (%)**	**n(%)**	**n (%)**
< -3	30 (20.5)	46 (28.8)	32 (21.6)	46 (29.3)
≥-3 to < -2	30 (20.5)	53 (33.1)	21 (14.2)	30 (19.1)
≥-2 to < -1	27 (18.5)	23 (14.4)	19 (12.8)	51(32.5)
≥-1 to 0	24 (16.4)	29 (18.1)	26 (17.6)	17 (10.8)
0 to ≤ 1	18 (12.3)	18 (11.3)	13 (8.8)	11 (7.0)
> 1 to ≤ 2	7 (4.8)	12 (7.5)	22 (14.9)	2 (1.3)
> 2 to ≤3	5 (3.4)	2 (1.3)	8 (5.4)	0 (0)
> 3	5 (3.4)	4 (2.5)	7 (4.7)	0 (0)

*Abbreviations*: *HAZ* height-for-age z-score, *WAZ* weight-for-age z-score, *WHZ* weight-for-height z-score, *WLZ* weight-for-length z-score, *MUACZ* mid-upper arm circumference z-score, *CI* confidence interval, *SD* standard deviation

The mean HAZ was calculated to be –1.4 (95%CI –1.8; –1.0) and the mean WHZ was –0.9 (95%CI –1.3; –0.5). Out of the 70 SAM patients, WHZ was calculated for 55 of the children and HAZ was calculated for 56 of the children (Table 2). Nearly 30% of the SAM patients were classified as wasted and stunted (WHZ < –2 and HAZ < –2).

Factors that were associated with SAM (odd ratio (OR), 95% CI) among the hospitalised children were being male (2.8; 2.7–3.0) and being ill with an infection 2 weeks prior to admission (2.1; 1.4–3.1). Increasing dietary diversity (0.7; 0.7–0,8), increasing HAZ (0.8; 0.8–0.8), secondary education for the mother (0.3; 0.3–0.4) and father (0.4; 0.3–0.6), employment of the father (0.4; 0.2–0.8), and being in the highest SE-index group (0.2; 0.1–0.4) were protective factors according to the bivariable analyses. In the adjusted model, male sex (4.6; 2.3–9.2) was more prominently associated with SAM, and improved dietary diversity (0.9; 0.7–1,0), higher father’s education (0.4; 0.4–0.5), and higher SE category (middle group 0.3;0.3–0.4, highest group 0.1; 0.0–1.3) were significantly associated with less SAM in the hospitalised children (Table 3).

**Table 3:** Factors associated with SAM in children aged 6 - 59 months

Variable	**SAM**			**Crude analysis**			Adjusted analysis		
	No (%)	Yes (%)	Total	OR	95% CI	p-value	OR	95% CI	p-value
**Inherent**									
**Sex**									
Female	83 (79.3)	33 (47.1)	116 (63.7)	ref			ref		
Male	31 (26.3)	35 (50.0)	66 (36.3)	2.84	2.67 - 3.01	**< 0.001**	4.62	2.30 - 9.16	**< 0.001**
**Age child**	-	-	-	1	0.97 - 1.03	0.863	-	-	-
**Immediate factors**									
**DDS7**	-	-	-	0.74	0.66 - 0.84	**< 0.001**	0.78	0.65 - 0.95	**0.012**
**HAZ**	-	-	-	0.83	0.83 - 0.83	**< 0.001**	1.02	0.91 - 1.13	0.767
**Hospital admission last 12 months?**									
No	79 (66.9)	35 (50.0)	114 (60.6)	ref			-	-	-
Yes	39 (33.1)	35 (50.0)	74 (39.4)	2.02	0.94 - 4.32	0.068	-	-	-
**Symptoms of diarrhoea, fever or cough two weeks prior to admission**									
No	63 (53.4)	25 (35.7)	88 (46.8)	ref			ref		
Yes	55 (46.6)	45 (64.3)	100 (53.2)	2.06	1.38 - 3.07	**< 0.001**	1.37	0.88 - 2.11	0.162
**Mothers education**									
No school	37 (31.4)	29 (41.4)	66 (35.1)	ref			ref		
Primary	44 (37.3)	29 (41.4)	73 (38.8)	0.84	0.45 - 1.56	0.584	1.5	0.51 - 4.39	0.454
Secondary	36 (30.51)	10 (14.3)	46 (24.5)	0.35	0.30 - 0.40	**< 0.001**	1.92	0.41 - 9.10	0.409
**Occupation mother**									
Unemployed	87 (73.7)	52 (74.3)	139 (73.9)	ref			-	-	-
Employed	31 (26.7)	17 (24.3)	17 (24.3)	0.92	0.38 - 2.23	0.849	-	-	-
**Fathers education**									
No school	28 (23.7)	14 (20.0)	42 (22.3)	ref			ref		
Primary	29 (24.6)	32 (45.7)	61 (31.5)	2.2	2.14 - 2.27	**< 0.001**	1.42	1.16 - 1.74	0.001
Secondary	58 (49.2)	12 (17.1)	70 (37.2)	0.41	0.28 - 0.60	**< 0.001**	0.42	0.37 - 0.48	**< 0.001**
**Fathers occupation**									
Unemployed	56 (47.5)	42 (60.0)	98 (52.1)	ref			ref		
Employed	59 (50.0)	19 (27.1)	78 (41.5)	0.43	0.22 - 0.84	**0.013**	1.41	0.59 - 3.37	0.433
**SE-index**									
Lowest index group	27 (23.7)	35 (50.0)	62 (33.7)	ref			ref		
Middle index group	36 (31.6)	25 (35.7)	61 (33.2)	0.54	0.27 - 1.05	0.07	0.3	0.24 - 0.37	**< 0.001**
Highest index group	51 (44.7)	10 (14.3)	61 (33.2)	0.15	0.06 - 0.38	**< 0.001**	0.11	0.01 - 1.29	**0.081**
**Food security**									
Insecure	49 (41.5)	35 (50.0)	84 (44.7)	ref			-	-	-
Secure	55 (46.6)	32 (45.7)	87 (46.3)	0.81	0.20 - 3.23	0.771	-	-	-

*Abbreviations*: *SAM* severe acute malnutrition, *OR* odds ratio, *CI* confidence interval, *DDS7* Dietary diversity score 7 days before admission, *HAZ* height-for-age z-score, *SE-index* socioeconomic index

## Discussion

The aim of this study was to investigate the dietary diversity, nutritional practices, frequencies of undernutrition, and factors associated with SAM in an inpatient paediatric population aged 6–59 months in Hawassa, Southern Ethiopia. This study found that SAM constituted a large part of the children’s conditions (33%) followed by respiratory tract infections (23%). Children suffering from SAM had lower dietary diversity compared to other ill children, and the diet could be described as a monotonous diet mainly based on cereals and grains. There was little to no consumption of animal products, except for dairy and some egg products. Both inherent biological factors (sex) and household factors (education and socioeconomics) were related to SAM.

High rates of stunting and wasting were found in this study indicating that hospitalised children largely are nutritionally deprived and that SAM is a common reason for admission. Out of the 70 SAM patients, 28% were classified as wasted and stunted (WHZ < –2 and HAZ < –2), meaning nearly a third of them were experiencing multiple anthropometric deficiencies at the same time. Stunting and wasting are closely related and can occur together in a population and at the same time in a child (referred to as concurrence) [14, 15]. Being wasted increases the risk of subsequent stunting, and children experiencing stunting and wasting have a higher risk of mortality, and even more so when they occur repeatedly or at the same time [16, 17]. Stunting increases in the age-group 5–23 months and peaks at age 24–35 months [1].

According to the Ethiopian Mini Demographic and Heath study (EMDHS) from 2019, there were high levels of undernutrition in the SNNPR [18]. They reported that 19.7% had WAZ below –2SD (mean –1SD), 36.3% of the children had HAZ below –2SD (mean –1.4SD) and 6.3% had WHZ below –2SD (mean –0.3 SD) [18]. The EMDHS is a household-based survey, whereas this study was hospital based. Up-scaling of CMAM has been more common in the last years in Ethiopia, and if the child has signs of appetite and is not sick they can be treated in an out-patient program. Thus, only the most serious cases with co-morbidity are admitted to hospitals, which was reflected in the anthropometric status we saw in this study. However, the high patient load in the hospitals regarding undernutrition may indicate that the community programs are not yet fully successful at preventing and treating these conditions.

In our study, males were found to have higher odds of SAM compared to females. The role of gender in the development and outcome of disease patterns is a complicated matter of hormones and genes as well as lifestyle, behavioural, and socioeconomic differences [19]. For SAM patients, several studies have suggested that there is no significant association between SAM and gender [20, 21]. Still, growth data suggest that boys are more likely to be stunted and wasted than girls and that boys experience being stunted and wasted at the same time more often than girls [16, 22, 23]. Higher HAZ was seen to have a significant protective association for SAM patients. Stunting represents chronic undernutrition, and a HAZ that is within the normal range and stable within one’s own trajectory indicates that the child is growing normally and has not experienced long-term growth deficits. However, it is possible to experience SAM and still have HAZ within the normal ranges. Also, by using attained growth data at only one time point we do not know if there has been severe growth faltering without crossing the <–2 threshold.

Regarding food diversity, our data suggest that there was a difference in mean scores between the two patient groups. Because appetite loss is a major reason for hospitalisation, some differences in dietary assessments were expected. The data obtained from the DDS showed that the majority of the children did not have a diverse diet. Half of the participants in our study experienced some degree of food insecurity. A study from the Bensa District in the Sidama region reported similar numbers, with 48.2% of households being food secure and 52.1% experiencing some degree of food insecurity. They concluded that children from food secure families received a more optimal complementary feeding when comparing them to children from families experiencing food insecurity [24]. Another study from the Boricha district in Sidama reported that 82% experienced food insecurity over a year and showed seasonal variations with higher numbers of food-insecure households in March (69%) compared to September (50%) and December (38%) [25].

Our dietary diversity questionnaire was extended to a 7-day recall because the majority of the patients had been hospitalised for longer than 24 hours and the recall would have been affected by the hospitalisation. The majority of the children (62%) had a score below four, but 38% of the children had a diet with four or more food groups. Other published data from the region suggest much lower percentages, but these are based on 24-hour recall. We cannot rule out the possibility of recall bias with a 7-day recall and social desirability bias in this population in this hospital-admitted group. However, they may have reported to the best of their ability, and the relatively high number of children with more diverse diets may reflect an attempt to feed the children and comply with recommendations. The EDHS from 2016 reported that only 14% of Ethiopian children had an adequately diverse diet, the proportion being 13% in the SNNPR [1]. Children admitted with SAM had a lower mean score in all the age groups, and a higher proportion of the children (75%) had a score below four, which indicates a diet that is not adequately diverse. It is worrying to see that not only is the mean dietary diversity score for the young age group too low, but also that the older children (24–59 months of age) do not reach this cut-off point.

The consumption of foods in this study was characterized by a low intake of animal-sourced foods together with a not-so-diverse diet. A report from UNICEF and GAIN about nutrient gaps in complementary feeding for children 6–23 months in Ethiopia reported that iron, zinc, calcium, iodine, and vitamin A were micronutrients of special concern in Ethiopia [26]. This study did not explore any biochemical data, and the food patterns were not analysed on an individual level. Thus, it is not possible to say anything specific about possible micronutrient deficiencies in these children.

Recent community-based studies from the Sidama Zone have reported parallel findings. In Aleta Wondo, a study showed that the mean DDS was 2.5 and only 12% of the children had met the minimum dietary requirements [27]*.* A study from Shebedino and Hula districts looked at the differences in DDS between the dry and wet seasons. Nearly 41% of the children met the minimum DDS, but this percentage dropped to 35% in the lean wet season. The mean DDS was 3.3 and 3.2 in the wet and dry seasons, respectively [28]. Thus, the literature points towards seasonal and geographical variability.

### Strengths and limitations

There are several limitations to this study. The cross-sectional design can never rule out the reverse causality of any associations found; for example, the low dietary diversity may be causing undernutrition, but may also be a result of undernutrition if undernutrition has caused poor appetite. The growth restriction may be due to other factors than what is measured at the point of data collection, such as intrauterine or early child development-related factors.

Also, by using a retrospective recall to collect information of diet and symptoms of a disease, there is always a chance for recall bias. We did not cover properly given supplementary feeding at the hospital in our assessment, and thus we may have underreported some foods given. However, our interest was the diversity of normal foods, not treatment foods. We know that standard treatment of SAM was implemented at the two hospitals, and three standard meals were given to other paediatric patients, and thus a more accurate presentation of the current diet of the children should have been presented.

Random and systematic errors can occur when taking anthropometric measurements. Random errors are unavoidable and can occur when measuring a child. Anthropometric measurements are dependent on the accuracy of the person doing the measuring, and accuracy is determined by their intra-rater reproducibility and how valid their measurements are compared to an ideal measurer. If the child moves or changes position, this can affect the measurements. Accuracy is also dependent on how well the staff reads the scale. Systematic errors can happen if the instruments are not calibrated. To minimize these possible errors, we trained the staff in taking anthropometric measurements, performed a review of the questionnaire and possible challenges, calibrated the instruments, and had supervisors following the process closely in the hospital.

Strengths of this study include a systematic description of diet and anthropometry among children hospitalised in public health facilities in Hawassa, Ethiopia.

## Conclusion

This study showed that hospitalised children in Hawassa had a high degree of morbidity, extreme poverty, and poor nutrition with low dietary diversity. Little research has been done with a focus on nutrition patterns and status among children in clinical settings compared to community-based studies. Because 35.8% of the children were wasted and 40.1% were stunted, further research should also address factors before admission, food service at the hospital, and therapeutic nutrition treatment for other patient groups than those with SAM.

## Supplementary Information

**Additional file 1.**

## Data Availability

The datasets used and/or analysed during the current study are available from the corresponding author on reasonable request.

## Abbreviations

CI: Confidence interval
CMAM: Community Management of Acute Malnutrition
DDS: Dietary Diversity Score
EDHS: Ethiopian Demographic and Health Survey
HAZ: Height-for-age z-score
HFIAS: Household food insecurity access scale
MUAC: Mid-upper arm circumference
MUACZ: Mid-upper arm circumference z-score
OR: Odds ratio
PCA: Principal component analysis
SAM: Severe acute malnutrition
SE-index: Socioeconomic index
SNNPR: Southern Nations, Nationalities, and People’s Region
WAZ: Weight-for-age z-score
WHO: World Health Organization
WHZ: Weight-for-height z-score
WLZ: Weight-for-length z-score
UNICEF: The United Nations Children’s Fund
