# Supplementary material for: Diet and nutritional status among hospitalised children in Hawassa, Southern Ethiopia
Source: BMC Pediatr. 2022 Jan 21;22:57. doi: 10.1186/s12887-022-03107-6 (PMC8781358; doi:10.1186/s12887-022-03107-6)
Supplement: Supplementary file 1 — Additional file 1. [file 12887_2022_3107_MOESM1_ESM.docx]

**Supplementary appendices**

**Calculations of z-scores**

For weight-for-age z-score (WAZ), height-for-age z-score (HAZ,) and weight-for height/length z-score (WHZ), the Zscore06 command was used. In addition, the igrowup_standard.ado macro for STATA was used to calculate mid-upper arm circumference z-score (MUACZ). Flagging of the z-scores followed the WHO flag system (Leroy, 2011; WHO):

- Height/length-for-age: < -6 or > +6
- Weight-for-age: <-6 or > +5
- Weight for length/height: < -5 or > +5
- (Mid-upper) Arm circumference-for-age z-score: < -5 or > +5

Z-scores that fell outside these thresholds were assessed individually, and if excluded, set to missing. Children were considered stunted, wasted or underweight if the z-scores for height-for-age, weight-for-age and weight-for-height were -2 SD from the median value in the WHO 2006 growth standard. If the z-scores were -3 SD they were classified as severely stunted, severely underweight and severely wasted. Having a MUAC z-score below -2 SD was defined to be malnourished and below -3 SD to be severely malnourished. When calculating the z-scores in STATA the Zscore06 takes into account measurements of recumbent length and height in the different age groups. For the simplicity of writing, only height-for-age z-scores (HAZ) and weight-for-height z-score (WHZ) will be used further in the article.

**Table 1 : Distribution of disease categories in the hospitalized children in Hawassa, 2020**

| **Variable** | **Adare Hospital**  **n = 73 (%)** | **Referral University Hospital**  **n = 115 (%)** | **Total**  **n = 188 (%)** |
| --- | --- | --- | --- |
| Respiratory tract infections | 26 (35.6) | 18 (15.7) | 44 (23.4) |
| Abdominal infections | 9 (12.3) | 5 (4.4) | 14 (7.5) |
| Severe acute malnutrition | 6 (8.2) | 57 (49.6) | 63 (33.5) |
| Other | 27 (37.0) | 23 (20.0) | 50 (26.6) |
| Missing | 5 (6.9) | 12 (10.4) | 17 (9.0) |

**Figure 1: Consumption of food groups for paediatric patients seven days pre-hospitalisation**
